# Approaching *Etuaptmumk* – introducing a consensus-based mixed method for health services research

**DOI:** 10.3402/ijch.v74.27438

**Published:** 2015-05-22

**Authors:** Susan Chatwood, Francois Paulette, Ross Baker, Astrid Eriksen, Ketil Lenert Hansen, Heidi Eriksen, Vanessa Hiratsuka, Josée Lavoie, Wendy Lou, Ian Mauro, James Orbinski, Nathalie Pabrum, Hanna Retallack, Adalsteinn Brown

**Affiliations:** 1 Institute for Circumpolar Health Research, Yellowknife, NT, Canada; 2 Institute of Medical Science, University of Toronto, Toronto, ON, Canada; 3 Stanton Territorial Health Authority, Elders Council, Yellowknife, NT, Canada; 4 Institute of Health Policy, Management and Evaluation, University of Toronto, Toronto, ON, Canada; 5 Centre for Sami Health Research, University of Tromsø, Tromsø, Norway; 6 Health Care Centre Utsjoki, Municipality of Utsjoki, Finland; 7 Southcentral Foundation, Anchorage, AL, USA; 8 MFN-Centre for Aboriginal Health Research, Faculty of Health Sciences, University of Manitoba, Winnipeg, MB, Canada; 9 Dalla Lana School of Public Health, University of Toronto, Toronto, ON, Canada; 10 Department of Geography, University of Winnipeg, Winnipeg, MB, Canada; 11 Balsillie School of International Affairs, Wilfrid Laurier University: Centre for International Governance Innovation, Waterloo, ON, Canada; 12 National Aboriginal Council of Midwives, Winnipeg, MB, Canada

**Keywords:** health systems stewardship, indigenous values, mixed methods

## Abstract

With the recognized need for health systems’ improvements in the circumpolar and indigenous context, there has been a call to expand the research agenda across all sectors influencing wellness and to recognize academic and indigenous knowledge through the research process. Despite being recognized as a distinct body of knowledge in international forums and across indigenous groups, examples of methods and theories based on indigenous knowledge are not well documented in academic texts or peer-reviewed literature on health systems. This paper describes the use of a consensus-based, mixed method with indigenous knowledge by an experienced group of researchers and indigenous knowledge holders who collaborated on a study that explored indigenous values underlying health systems stewardship. The method is built on the principles of *Etuaptmumk* or two-eyed seeing, which aim to respond to and resolve the inherent conflicts between indigenous ways of knowing and the scientific inquiry that informs the evidence base in health care. Mixed methods’ frameworks appear to provide a framing suitable for research questions that require data from indigenous knowledge sources and western knowledge. The nominal consensus method, as a western paradigm, was found to be responsive to embedding of indigenous knowledge and allowed space to express multiple perspectives and reach consensus on the question at hand. Further utilization and critical evaluation of this mixed methodology with indigenous knowledge are required.

The provision of health services in the circumpolar context has proven to be challenging. Common reasons cited for these challenges have included human resource issues, the difficulties of accessing remote areas without roads, the high burden of disease, historical trauma, and health services’ lack of cultural responsiveness to indigenous people (1). Throughout the circumpolar regions, governments and stakeholders who oversee health services delivery are working to improve the cultural components and responsiveness of the health care system and related policies. At the international level, declarations such as the United Nations Declaration on the Rights of indigenous Peoples have recognized the rights of indigenous peoples to maintain access to their traditional medicines and health practices, including the conservation of vital medicinal plants, animals and minerals. The UN declaration also calls for the right to access, without discrimination, all social and health services (2). Circumpolar nations have agreed to the terms of these declarations (2,3); however, there is a lag in the evidence base required to inform improvements in care.

With the recognized need for health systems improvements in the circumpolar and indigenous context, there has been a call to expand the research agenda across all sectors influencing wellness and to recognize academic and indigenous knowledge through the research process (4). Such a research approach requires systematic and holistic approaches to indigenous and western ways of knowing in order to gain insight into health systems’ strengths and adaptations applicable in the circumpolar setting (1,5).

This paper describes the use of a mixed methods framework by an experienced group of researchers and indigenous knowledge holders who collaborated on a study that explored indigenous values underlying health systems stewardship (the findings of this study are published elsewhere). In this paper, we will describe the components of a consensus-based, mixed method with indigenous knowledge. We will first describe the context for indigenous knowledge as it is applied in health research. Secondly, we will highlight the scholarship and approach underlying mixed methods. Thirdly, the applications of the mixed methodology with indigenous knowledge recognized as a distinct paradigm will be highlighted; finally, we will provide an example of a mixed method approach with indigenous knowledge embedded in a modified nominal design. The conclusions will highlight the strengths and challenges of the mixed methods approach, with recommendations of areas for further methodological development and study.

## Context of indigenous knowledge

The development of indigenous knowledge systems covering all aspects of life, such as community wellness and management of the natural environment, has been a matter of survival to the peoples who generated these systems. While these knowledge systems mean different things to different people, overall, such knowledge systems are cumulative, representing generations of experience, careful observation and trial-and-error learning. These bodies of knowledge hold significant social, cultural and scientific value, embracing both the content of the knowledge as well as traditional forms of expressing it (6,7). While there is not one indigenous body that agrees on a definition of traditional knowledge at the international level, the World Intellectual Property Organization has stated that:

Indigenous knowledge in a general sense embraces the content of knowledge itself as well as traditional cultural expressions and in the narrow sense refers to knowledge resulting from intellectual activity in a traditional context, and includes know-how, practices, skills, and innovations. (8)

In addition to the international context for indigenous knowledge, there are also definitions specific to nations. For Inuit, the term *Qaujimajatuqangit* (IQ) captures elements of traditional knowledge. It has been translated into English as “that which tries to capture past, present and future experience, knowledge and values of the Inuit” (9). The Sami use the concept of *árbediehtu*, a North Sami term containing two interrelated parts: *diehtu* “knowledge” and *árbi* “heritage/ inheritance”. This definition clarifies knowledge as both the information and the process, and emphasizes different ways to gain, achieve or acquire knowledge (10,11). In addition to the importance of indigenous knowledge within nations, governments also recognize the importance of these knowledge bases in their decision-making and operations. The Nunavut Government has highlighted that it will use IQ as its foundation, and the government of the Northwest Territories recognizes that Aboriginal Traditional Knowledge is a valid and essential source of information (9,12).

### Indigenous knowledge and research

In recent decades, academic study of indigenous knowledge has been primarily conducted within the social sciences disciplines, and this perspective of indigenous knowledge has dominated the peer-reviewed and scientific literature. However, the academic community only provides a limited view of the depth of knowledge and is often a translation of traditional knowledge. Porsanger, for example, differentiates between the concepts of “indigenous research” and “research on, with, and about indigenous peoples”. Indigenous research here being defined as that which is built on indigenous theorizing and knowledge. She distinguishes indigenous research from research that is conducted by outside researchers on their own terms and for their own purposes, regardless of the level of collaboration and respect (10). Ánde Somby, a Sami law scholar explains how approaches to indigenous research are a matter of “*re-socializing*”, that is, “coming to know our limitations and understand our place in our own society on our own terms, not to show our belonging to others, nor to defend our understandings, but to gain strength and intellectual independence” (10).

Despite being recognized as a distinct body of knowledge in international forums and across indigenous groups, examples of methods and theories based on indigenous knowledge are not well documented in academic texts or peer-reviewed literature on health systems. As a result, indigenous knowledge and indigenous research methods are often either not accessible, or not perceived to be valid sources of evidence in many academic communities (13,14). As such, academics and decision-makers do not have clear direction on how this knowledge can be accessed as a form of evidence to inform the generation of knowledge and decision-making, specifically in the field of indigenous health. In many instances, indigenous knowledge holders are underutilized, and their expertise is not applied in health systems research.

### Etuaptmumk/two-eyed seeing

In the shifting climate of repatriation and reconciliation, there has been a call to “explore, value, and use indigenous knowledge and methods on an equal footing with western knowledge and methods, and for integrating indigenous and western methods when appropriate” (14,15). To this end, the principles of *Etuaptmumk*/two-eyed seeing have been presented as guiding principles for integrative science that builds on indigenous knowledge and methods.

These principles cover all aspects of our lives including social, economic and environmental. It is about life: what you do, what kind of responsibilities you have, how you should live while on Earth (16). The principles have been used to guide studies in environmental sciences, health, education, social justice and discussions for cultural competency (16,17). These principles also serve as a foundation for the business case for the Canadian Institutes for Health Research, Institute for Aboriginal Peoples’ Health (17), and strategies such as the DRAFT Recovery Strategy for the American Eel in Ontario (18).

When applied, the principles of *Etuaptmumk/*two-eyed seeing aim to respond to and resolve the inherent conflicts between indigenous ways of knowing and the scientific inquiry that informs the evidence base in health care. While the principles of two-eyed seeing are gaining recognition, there is a need for further development of research methods that are responsive to the principles and approaches of indigenous knowledge holders and academic scholars who are working primarily in frameworks responsive to western knowledge.

## Mixed methods

The popularity of mixed methods has been well documented over the past decade (19). With the complimentary utilization of quantitative and qualitative data, the approach has been described as being a more intuitive approach to inquiry. A composite definition for mixed methods highlights the key aspects of the approach:

Mixed methods research is the type of research in which a researcher or team of researchers combines elements of qualitative and quantitative research approaches (e.g., use of qualitative and quantitative viewpoints, data collection, analysis, inference techniques) for the purposes of breadth and depth of understanding and corroboration. (20)

Greene's definition of mixed methods follows the principles of two-eyed seeing:

… that actively invites us to participate in dialogue about multiple ways of seeing and hearing, multiple ways of making sense of the social world, and multiple standpoints on what is important and to be valued and cherished. (21)

Over the years, there have been significant developments in both the definition and methodologies associated with mixed methods. The approach combines methods, a philosophy and a research design orientation. In practice, the researcher collects and analyses both qualitative and quantitative data, mixes the two forms of data, gives priority to one source of data, uses procedures in a single study, frames the study in philosophical worldviews and combines the procedures into a specific research design (19). The worldview associated with mixed methods has received much attention from mixed methods researchers, and some debate around research paradigms has ensued (19,22).

The inclusion of indigenous knowledge and scholarship in the field of inquiry, with the framing as a mixed method, introduces another research paradigm in that it honours a common set of beliefs, values and assumptions that a community holds in common. Similar to quantitative or qualitative paradigms, indigenous knowledge is a perspective critical to enhance the breadth of understanding in the field of health research inquiry. The mixed methods approach respects the recognized need for a reciprocal, mutually respectful, dialogic relationship between philosophical frameworks and methodological decisions (23). This design lends well to knowledge generation between paradigms and opens a pathway for consideration by academic researchers and indigenous knowledge holders.

**Fig. 1:**
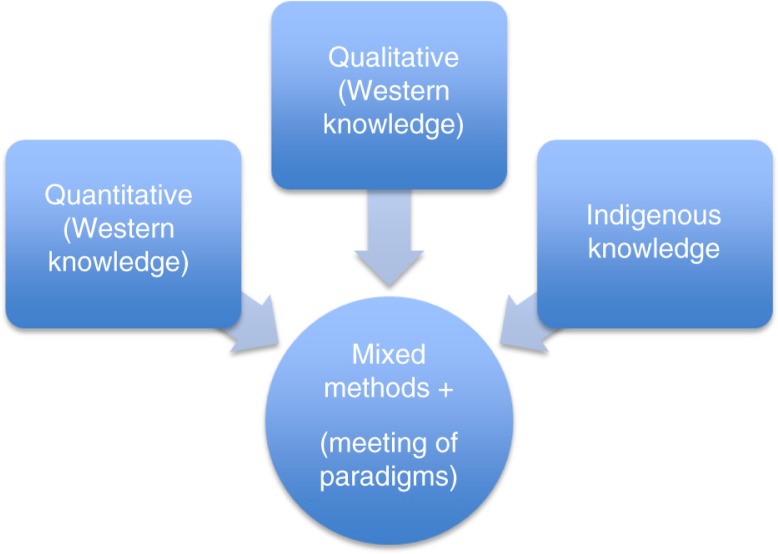
Mixed methods, western and indigenous knowledge.

Figure 1 provides a visual on how indigenous knowledge might position, relate to, and be included as an accepted research paradigm (or research culture) within a more widely accepted construct that inform our understandings around health and wellness in academic forums.

### Mixed methods and indigenous knowledge

While the majority of mixed methods literature is grounded in western ways of knowing, both highlighted the benefits of mixing methods to clarify the relationship between western research and indigenous ways of knowing so that more appropriate theories, practices and relations can be developed for their inter-relation. The process in itself has been argued to be a vehicle to decolonize – and reconcile – indigenous and western approaches (13,24). Healey and Tagak (25) and Simonds and Christopher (14) also called for the attention of indigenous knowledge in mixed methods research with a focus on the inclusivity of relational paradigms, and options as to how this may be achieved in research practice. This paper adds to current knowledge by exploring the methodological considerations in approaching mixed methods research with western and indigenous knowledge as distinct paradigms, and how this method can support inquiry in the area of health services research.

## Application of the mixed methodology with indigenous knowledge

We explored the values underlying health systems stewardship through a collaborative consensus-based approach with indigenous scholars and knowledge holders. An embedded, transformative, emergent mixed methods design was used in this study (19). The embedded design entails the collection of one type of data within a design framework associated with the other type of data.

The research question focused on identifying indigenous values that underlie health systems stewardship. In this case, the utilization of experts and data sources exclusive to health systems research would potentially have limited the scope of the findings when applied in an indigenous context. Given the social political context within the circumpolar regions, the need to include and complement the inquiry related to health systems stewardship, with indigenous knowledge rooted in traditional methods was recognized.

**Fig. 2:**
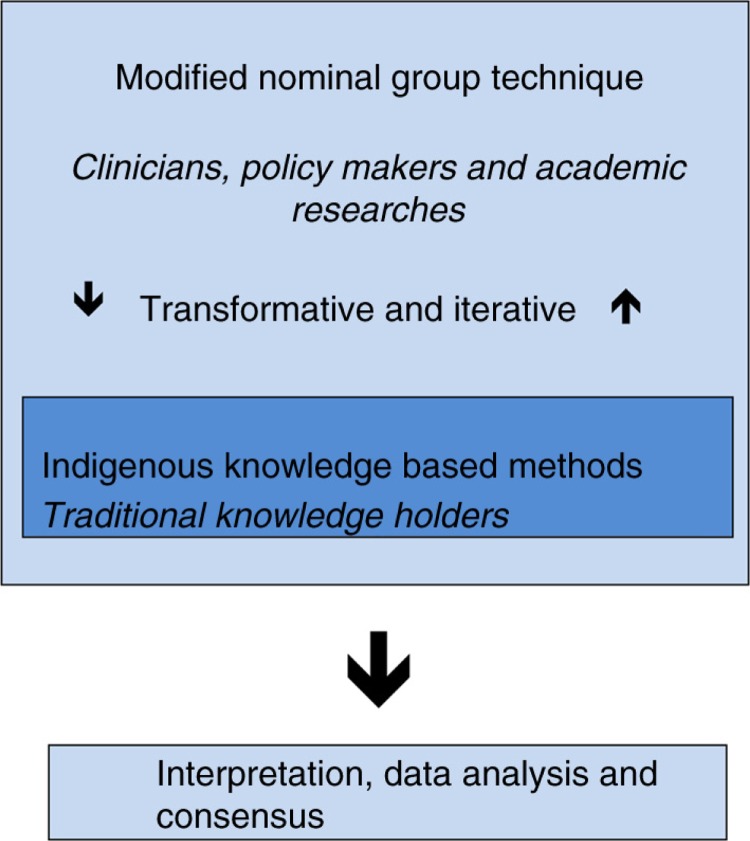
Consensus process.

The mixed methods approach included western knowledge and indigenous knowledge and strived to bridge a process that was relevant to health systems scholarship and processes respectful of indigenous scholarship. As such, the embedded approach allows for a supplemental data set that captures indigenous knowledge within a larger design that is more familiar to management sciences. A transformative approach was selected so that the study could be flexible, and also respectful of indigenous peoples and their knowledge (19). Figure 2 highlights the consensus process and context for the knowledge bases and paradigms.

### Consensus processes

Consensus methods are used in health care when there is a lack of information, or conflicting information on a health topic and a structured environment is required for decision-making (26). The Delphi and nominal group approaches have undergone extensive use and development in the areas of health and medicine. While there are some challenges to the approach, overall there is agreement that the format process and outputs have successes (27–29). In circumpolar regions with lagging research infrastructure and small populations, it is not uncommon for the evidence base with findings applicable to northern and indigenous populations to be lagging. Conversely, there is a wealth of expertise in indigenous approaches to wellness and clinical context in circumpolar regions. In this setting, the ability to use a structured process to address problems in health service provision or indicator development for further study has its appeal and promise. In this instance, the authors first discussed the approach and felt it would adapt well to, and accommodate indigenous knowledge and approaches to sharing expertise and knowledge.

**Table I:** Four phases of nominal group process

*Phase 1 – Independent synthesis*
Participants were asked to work independently and to identify between ten and twelve values that were written on cards. This component was done independently to maintain an anonymous process and to allow each participant to express views without influence.
*Phase 2 – Sharing and grouping themes*
The values were then shared by placing the cards on a wall to facilitate group discussion. Each participant put forward six values and the combined group's values were placed for viewing. A facilitated and interactive process with discussion between participants allowed for values to be grouped into unnamed themes (overarching values), and discussion around the themes and allocation of values took place in groups. Following this exercise, participants either placed their remaining cards under existing themes or placed them aside to be assigned by the group or within a new theme. Further discussion explored the meaning of the values and themes.
*Phase 3 – Identification of values*
The third phase entailed assigning a description to the value groupings. This work was done individually or in small groups. Upon completion of the individual or small-group work, information was shared in a large group session, and discussion around the descriptions took place. The dialogue provided further opportunities to share perspectives on themes and to clarify meanings. Consensus was further built through this process.
*Phase 4 – Description of values*
Each value description shared in the face-to-face session was recorded on a spreadsheet and put in a shared on-line workspace for all participants to view. The value descriptions were then summarized through written feedback and phone conversations, and a heading was assigned to each value. This component was carried out by email collaboratively after the face-to-face workshop.

While consensus methods have been used alongside other methods to enhance the robustness of research findings in a variety of health care settings (30,31), the processes have all been framed within paradigms of western knowledge and associated perceptions of health and wellness. In response to the need to be more responsive to indigenous groups and the need to include indigenous knowledge, mixed methodologies and the associated frameworks provide a process to engage across paradigms and build on the strengths of western knowledge with that of indigenous knowledge via a structured process.

### Nominal group process

The nominal group process is a process that was developed to provide a process for obtaining qualitative information for the purposes of health planning (32). This method has been used to explore challenges in health, social service, education, government and industry (27). The nominal process allows for the participants to engage in the development of the question, then through a series of rounds contribute their ideas to a composite list, which is then evaluated individually and as a group, with repeated rounds that allow for clarification and reflection. In this study, the process was endorsed by all participants and was responsive to both western approaches of a workshop-type format with cue cards and structured processes, and indigenous approaches through expression of knowledge through stories, film and ceremony that were facilitated and structured within indigenous ways of knowing.

As with any consensus process, high levels of effectiveness were strived for through attention to the selection of participants, how the information was presented, how the information was structured and the method of synthesizing (28). Table I describes the four phases of the nominal group process. The subsequent sections of this paper describe how the nominal group process was carried out as a component of a mixed method that enabled expression of indigenous knowledge.

### Embedded traditional knowledge in nominal group

The indigenous context of circumpolar health systems was highlighted and explored through the use of indigenous knowledge shared by participants. Each participant was asked to consider the research question prior to the workshop and invited to bring “data” or share experiences they felt would translate their experiences based on indigenous knowledge to the group. Through this process, indigenous knowledge was complementary to the consensus exercise when shared through photographs and films, and stories alongside the facilitated process.

The research process was iterative and participants provided input on design, implementation and analysis. The transformative design was emergent to allow investigators to adjust their interactions as required and to allow for the expression of methods more conducive to indigenous knowledge or management science.

The next section describes how a modified nominal design was carried out with indigenous knowledge embedded. Overall, the process was iterative with adaptations as required, thus is not intended to be prescriptive to all settings; however, it does provide an example of process adaptations that allow consensus processes to build on indigenous knowledge.

### Modified nominal group technique components described with indigenous knowledge embedded

#### Selection of question

The purpose of this exercise was to explore the indigenous values underlying health systems stewardship in circumpolar regions. Stewardship has been described as the “careful and responsible management of the well-being of the population” and is the “very essence of good government” (33). It has been summarized to be a function of governments responsible for the welfare of populations and concerned about the trust and legitimacy with which its activities are viewed by the general public (34). The World Health Organization in the World Health Report 2000, highlighted stewardship as one of the four main functions of the health system (along with financing, creating and managing resources, and service delivery) and is an appropriate basis on which to reconfigure the health system (35). A systematic review of the literature captured six generic stewardship functions including: strategy formulation and policy development, intersectional collaboration and action, health system governance and accountability, attention to system design, health system regulation, intelligence (data and analysis) generation (36).

The question had emerged from a prior workshop that explored priorities for a collaborative research agenda in circumpolar regions (4). It was felt that it was important to understand how the health system was managed, and that a stewardship framework would be responsive to northern environments with numerous actors reconciling competing demands for limited resources. Stewardship is also felt to be responsive to the more holistic views of health and wellness commonly held in the north. The approach of first exploring underlying indigenous values was felt to be formative in understanding what might be important, and context setting, for good stewardship in jurisdictions where we see increasing authority, or shared oversight, for health systems being taken on by indigenous governments and stakeholders. The participants had the opportunity to review background information on the topic and consider the question and scope of health systems stewardship prior to the meeting.

At the outset of the group meeting, the scope of values and health systems stewardship in relation to existing frameworks was discussed (36). There was full agreement that the question had a high level of relevancy and participants had the opportunity to share information they felt was relevant and supported the question in the international and multination context. This sharing was done through narratives and photos. If there had not been agreement on the question, the question would have been modified until agreement was obtained.

#### Participants

The structure of the nominal groups aims to maximize the strengths of having experts consider an issue (26). This approach requires engaging traditional knowledge holders, clinicians and policy makers as participants (37). indigenous knowledge keepers are bound by complex systems and protocols that vary between cultural groups. Thus, the selection criterion for knowledge holders was not pre-determined by one set of criteria. Instead, core members were invited to participate in the panel and in turn the group who was invited was able to nominate additional participants.

The indigenous knowledge embedded in this design was conveyed in films, photographs and stories. These processes are frequently used to share indigenous knowledge, to enable communities to document their strengths and concerns and thus to promote critical dialogue (38,39). The process by which indigenous knowledge was shared was not prescribed by the process, but the norms and standards of respective indigenous knowledge keepers. There was also crossover with subject expertise in the clinical realm, policy and academia. This crossover occurred within and between participants.

#### Facilitation and co-leadership

As a participant, the nominal group leader must also have subject-matter expertise (27). The nominal group leader facilitated the definition of the problem, and overall process design, determining when each step in the nominal group process had been completed and deciding when agreement had been reached. The process was co-facilitated by a holder of traditional knowledge, who provided direction on the timing and process to engage in activities related to the expression of indigenous knowledge. Co-facilitating the iterative format requires the ability to move between indigenous knowledge and consensus methods. This approach requires expertise in facilitation techniques and co-leadership between academic and indigenous knowledge holders.

#### Location setting

Indigenous peoples’ knowledge is grounded in deep understandings of the people and the land. Knowledge is passed on through oral traditions and is measured against more recent experiences. In research rooted in the values and traditions of indigenous peoples, traditional settings have been found conducive to knowledge sharing in approaching and respecting indigenous paradigms (40). To this end, the workshop was based at a fly-in lodge in a northern region of Canada. The setting aimed to be reflective of indigenous and western knowledge (consensus methods) and to allow space for both research methods to be expressed. Participants had the opportunity to move between lecture settings and land-based activities, and to build trust and respect though the sharing of indigenous knowledge and local ceremony.

Through the mixed method of consensus with embedded participatory data, nine values were identified and described: humanity, cultural responsiveness, teaching, nourishment, community voice, kinship, respect, holism and empowerment. The descriptions of each value and relationship to health systems stewardship are described by the authors elsewhere.

#### Dissemination

It was recognized that the outputs of study required approaches that would be respectful of the paradigms within the mixed method approach. This paper represents the application of findings in an academic paper that explores the mixed methods process. Recognizing that film-based and narrative approaches are more conducive to capturing traditional knowledge, team members with expertise in transferring traditional knowledge through media prepared a film (41). The mixed-media approach to dissemination enables us to reach a large number of stakeholders. Publishing the proceedings and outcomes in film format allowed expression of the experience of a participatory process. Turning the camera on the process articulated the humanity of the participants and provided a respectful medium to capture the connections to values and histories.

## Discussion

This paper describes a consensus-based mixed method with indigenous knowledge that can be used in bridging uncertainty in health services research, and specifically in areas where indigenous populations are represented. The consensus-based, iterative and transformative process enabled a rich, empowering and relationship-building experience that showed potential for informing further health systems development in circumpolar regions. The approach creates opportunities to address important questions during times of reconciliation and repatriation of indigenous peoples’ rights in circumpolar nations. The ability to integrate methods and build on multiple knowledge bases, and scientific methods, opens doors to a methodological approach that is supportive of *Etuaptmumk* or two-eyed seeing.

The use of mixed methods with indigenous knowledge will allow researchers, stewards and community leaders to consider perspectives that are not well captured by traditional academic approaches, and to disseminate findings to broad audiences in academic and community settings, thereby facilitating better knowledge exchange and greater opportunities for implementation. The mixed methods were seen to be applicable both within communities and across nations as a basis for international study.

We are all affected around the globe, sometimes we are working in isolation, sometimes we are duplicating work and we need to be continuing to be coming together to find out what is needed, what is missing and what is done and how we can find solutions.

The facilitated consensus-based, transformative and iterative approach provided spaces for expressing indigenous knowledge and academic approaches. This proved to be a rich and moving experience for participants who had the opportunity to contribute to the development of the consensus process and inclusion of indigenous knowledge. The mixed method allows space for indigenous scholars to move between paradigms of western and indigenous knowledge and minimize the internal conflict that can emerge. Many of the workshop participants said that discussing values in a constructive and trusting environment had a positive impact on them.

The first thing that I come away with is I am not alone in being from an indigenous people, serving them … I am not alone in struggling to use methodologies that do not feel appropriate and seeking to re-invent or re-design those methodologies …

### Applicability

Many health care settings have recognized the need for enhancement of cultural relevancy of programmes and services for indigenous peoples (42). The methodology presented in this paper may aid in bringing consensus in areas where direction is required to improve programmes and promote system innovation. This approach offers a structured approach to shared solutions in situations where the peer-reviewed literature is lacking, yet expertise of indigenous knowledge holders and health systems stakeholders are prominent.

We have seen that consensus methods based on the knowledge bases of academics, managers, clinicians and end users are well established and widely published and applied in health care settings. Building on the strengths of the consensus approach, the addition of clearly defined contributions of indigenous knowledge holders provides an opportunity to advance knowledge and its applications for health systems improvements.

At all levels of government, it is more common that we see indigenous knowledge informing health and environmental related policy, and decision-making (9,43). Globally, there are escalating pressures on indigenous populations and identified needs for dialogue around issues such as climate change (44), resource development, protection of traditional knowledge (8,45), and access and traditional use of traditional medicine (46). The process described in this paper facilitated consensus in an international indigenous group. In this exercise, the experience was conductive to knowledge sharing and consensus building between the international experiences of indigenous groups across four nations (U.S., Canada, Norway and Finland) and four indigenous groups (Sami, First Nations, Inuit and Métis). The successes of this exercise demonstrate potential for applications in other international settings and regions.

### Nominal consensus techniques and indigenous knowledge

The structure of the nominal consensus process, imbedded indigenous knowledge and co-facilitation maximized the uptake of expertise from participants and minimized the potential of one personal or professional perspective dominating the process. The process created opportunities for indigenous scholars to be investigators and recognized for knowledge and expertise. This was seen to be of upmost importance in a dialogue inclusive and western and indigenous knowledge.

One challenge that emerged was the use of language in the multilingual groups. Where English was the common language, it was used for group activities. However, the limitations were recognized, and when possible the use of indigenous languages in these exercises would be optimal. An approach to consider in future exercises might be to have multiple participants representing language groups, who can hold breakout sessions according to language, with reporting back in shared language of group.

It is so hard … to stay true to some of the intentions because our (indigenous) language shapes and changes the essence of how we share things

Research groups utilizing the “mixed method” approach must have mutual respect for approaches and agreement on the knowledge paradigms are worthy of combining for the research question at hand. Thomas Kuhn popularized the idea of a paradigm and highlighted that it was a general concept that included a group of researchers having a common education and an agreement on “exemplars” of high-quality research or thinking (47).

In the context of mixed methods research, Johnson et al. (20) emphasize that a *research paradigm* (or research culture) is a set of beliefs (ontological, epistemological axiological, aesthetic and methodological), values and assumptions that a community of researchers has in common regarding the nature and conduct of research. In this context, they argue that there are three major research paradigms: qualitative research, quantitative research *and* mixed methods research (20). This finding also complements the writing of David Morgan who proposed that a paradigm can represent the shared beliefs of a research field (46). He goes further to highlight this perspective building on the work of Kuhn's view around communities of practice, and transitioning the focus from research paradigms to instead a focus on disciplinary matrix to summarize forms of groups consensus (47,48).

Further study on the communities of practice engaged in indigenous health research and the approaches utilized to enhance the inclusion of indigenous knowledge holders are required to advance the understanding beyond exclusive attention to indigenous and western paradigms. In the example highlighted, participants in the exercise came from a shared community of scholars and knowledge holders collectively interested in broadening approaches to understanding health systems improvements that are responsive to indigenous peoples. In this instance where indigenous knowledge was embedded in a nominal consensus method, a high level of trust and understanding of respective ways of knowing was present. In the end, the synergies between participants in the consensus exercise was a formative element of success.

## Conclusions

Where mixed methods frameworks have up-front philosophical assumptions, as well as methods of inquiry, the methods appear to provide a framing suitable for research questions that require data from indigenous knowledge sources and western knowledge. The nominal consensus method, as a western paradigm, was found to be responsive to embedding of indigenous knowledge and allowing space to express multiple perspectives and reach consensus on the question at hand.

Further utilization and critical evaluation of this mixed methodology with indigenous knowledge are required to advance the typology of the mixed method paradigm beyond qualitative and quantitative paradigms, and give further consideration to indigenous knowledge in mixed methods. This will inform respectful research collaborations, innovative solutions and knowledge to inform health systems improvements requiring the strengths of knowledge generated by two-eyed seeing.

The approach was found to be feasible for use in indigenous populations and where traditional knowledge is required to complement qualitative or quantitative studies and data. The nominal consensus approach was found to be beneficial in the circumpolar context where data for research can be limited due to population size, infrastructure and human resource limitations.
